# Neural spike prediction based on spreading activation

**DOI:** 10.1186/1471-2202-15-S1-P7

**Published:** 2014-07-21

**Authors:** Tielin Zhang, Yi Zeng, Bo Xu

**Affiliations:** 1 Institute of Automation, Chinese Academy of Sciences, Beijing, China; 2 University of Chinese Academy of Sciences, Beijing, China

## 

For each neuron in a neural network, its behavior does not only be decided by its own property, but also very relevant to its contexts (e.g. other neurons in the same network). Hence, effective prediction of neural spike activities in a network context requires at least the following three major efforts: (1) Response prediction of a single neuron towards a stimulus, (2) Obtaining the detailed network structure, with synapse information among neurons, (3) Modeling signal transmission based on the neural network.

For the first effort (e.g. single neuron response modeling towards a stimulus), many models can be applied, and in this study, we adopt the Poisson spike generator to construct the model [1]. This model receives a stimulus, and generates a response (a Boolean value on output a spike or not) through three processing steps, namely, linear filtering, static nonlinearity, and spike generation judgment [1]. For the second effort (neural network structure construction), the network structure is typically based on brain slicing and reconstruction with nanoscale imaging. Neural pathway prediction methods can also be used to generate the structure of the neural network.

As for the third effort, the modeling of the signal transmission process is designed to be based on the first two efforts. In addition, the followings should be considered in the modeling process: (1) when reaching the soma, the voltages will be reduced during the signal transmission process from synapses. (2) The voltage at the soma is sometimes a collective contribution from multiple neurons. Based on these two considerations, we propose to model the signal transmission process and predict possible neural spikes based on the spreading activation theory [2].

Assume a specific neuron (denoted as *n1*) is connected with *N* neurons in the network and its action potential is *V*, the post synaptic neurons of *n1* receive transmitted signals from *n1*. When one synaptic transmission is done and the signal reaches the post synaptic soma, its contribution to this soma is around 5mv [3]. The overall contribution to the voltage of the soma is represented as *P*=N*5mv, which is obtained by summing up all the contribution from each of the post synaptic potential, while P is used as the stimulus to generate next action potentials. Each of the potential *P* for the *N* neurons that connects to a specific neuron can be obtained through the upper calculation process. Having the structure of the neural network, we predict the neuron which owns the largest value of P will generate a spike.

**Figure 1:**
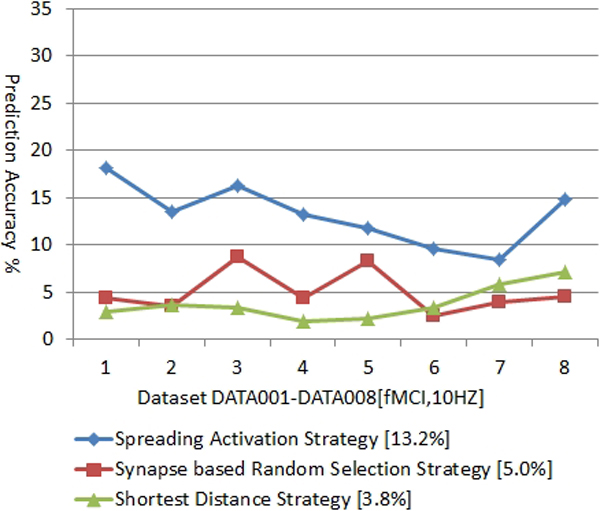
Neural Spike Prediction Accuracy based on Different Strategies

In order to validate the proposed method, the data from the rat hippocampus CA3 pyramidal cell layer using functional Multineuron Calcium Imaging (fMCI) is used (Including 8 datasets, and each of them records spike activities for 62 to 226 neurons. The datasets were pictured with the frequency of 10Hz [4,5]). Since the time slot during two neighborhood pictures is 100ms, signal transmissions may have done for several rounds. Hence, iterations of the spreading activation process are needed. The spike prediction accuracy for each of the dataset is shown in Figure 1.

As a comparative study, we introduce two alternative strategies, namely the shortest distance strategy (the neuron which owns the shortest distance compared to other post synaptic neurons will be fired), and the synapse based random selection strategy (randomly select a neuron from the set of post synaptic neurons). As shown in Figure 1, the spreading activation strategy outperforms other two strategies and the average prediction accuracy on 8 datasets is around 13.2% (the average prediction accuracy for shortest distance strategy is 3.8%, while the synapse based random selection strategy is 5.0%). The validation shows that the proposed spreading activation strategy is potentially effective for predicting neural spikes in the neural network.
